# *Thermus thermophilus* Strains Active in Purine Nucleoside Synthesis

**DOI:** 10.3390/molecules14031279

**Published:** 2009-03-24

**Authors:** Marcos Almendros, José-Vicente Sinisterra Gago, José Berenguer Carlos

**Affiliations:** 1 Biotransformations Group. Servicio de Biotransformaciones Industriales. Parque Científico de Madrid. C/ Santiago Grisolia nº 2. 28760 Tres Cantos. Madrid, Spain; 2 Centro de Biología Molecular *Severo Ochoa*, UAM 28049 Cantoblanco. Madrid. Spain.; 3 Department of Organic & Pharmaceutical Chemistry. Faculty of Pharmacy. Universidad Complutense, 28040 Madrid, Spain

**Keywords:** Biocatalysis, Nucleoside synthesis, *Thermus thermophilus*.

## Abstract

Several strains of *Thermus thermophilus* were tested in order to detect purine nucleoside synthase activity using pyrimidine nucleosides as the sugar-donor and adenine or hypoxanthine as bases. High productivity values (t =1 hr) were obtained while completely avoiding adenosine-deaminase degradation of the products. *N*-2-deoxy-ribosyltransferase activity is described for the first time in hyperthermophilic bacteria.

## Introduction

Thermophiles are a group of microorganisms that grow under extreme temperature conditions. These microorganisms offer useful enzymes to expand the range of reaction conditions suitable for biocatalysis. In this way some interesting enzymes have been described such as esterases, lipases, proteases, alcohol dehydrogenases, etc. [1,2]. Nucleoside analogues are labile and polyfunctional molecules which chemical synthesis requires several protection/deprotection steps [3]. These compounds have a wide range of uses, mainly as antiviral or antitumoral drugs, but also in the treatment of hypertension or inflammatory processes. The one-pot synthesis using nucleoside phosphorylases (NPs) or nucleoside 2’-deoxyribosyltransferases (NdRTs) are an alternative to the chemical synthesis [3,4,5]. 

**Scheme 1:**
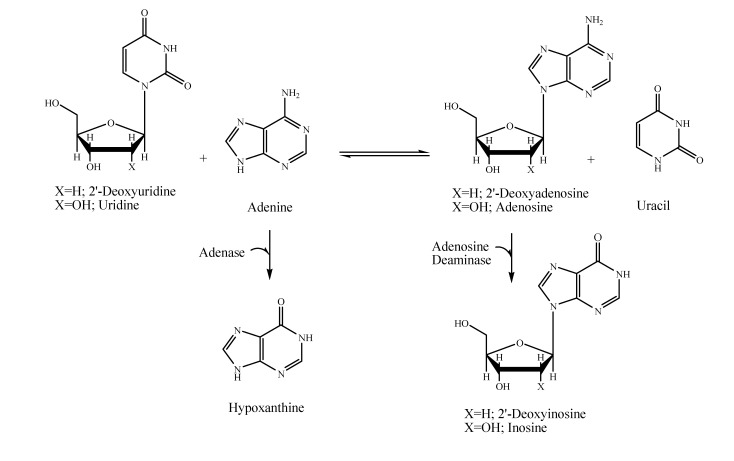
Reaction pathways of the adenine nucleoside synthesis and degradation of adenine and adenine nucleosides by *ADA.*

Several mesophile microorganisms have been identified as active for purine nucleoside synthesis, giving relatively good yield values after short reaction times [5,6]. However, the adenosine degradation by *adenosine-deaminase* (*ADA*) still remains as a problem for many of them (Scheme 1). In this work, we identify several strains of *Thermus thermophilus* capable of reaching high yield values, overcoming the *ADA* problem. 

**Scheme 2:**
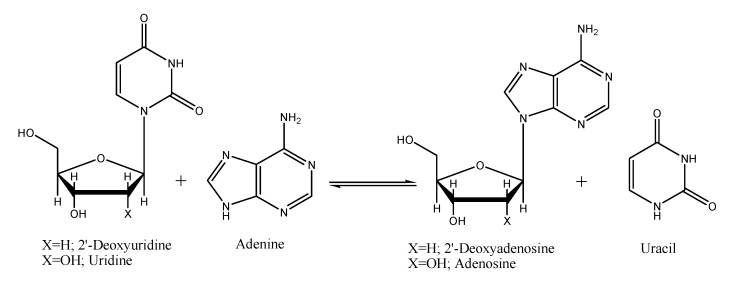
One-pot enzymatic synthesis of adenine nucleosides using uridine or 2’-deoxyuridine as donors of sugar moiety.

## Results and Discussion

The reactions of 2’-deoxyuridine (X =H) or uridine (X =OH) with hypoxanthine were tested. These reactions yield 2’-deoxyinosine (X =H) or inosine (X =OH), respectively (Scheme 2). Different *Thermus thermophilus* strains - cultured in optimum conditions - were tested. Better yields and productivity values were obtained with 2’-deoxyuridine (dUrd) than in the case of uridine (Urd) (Table 1). These results seem to indicate that one very active nucleoside 2’-deoxyribosyltransferase or one thymidine nucleoside phosphorylase plus one purine nucleoside phosphorylase are present in these strains. 

**Table 1:** Yield and productivity values in the reaction dUrd or Urd + hypoxanthine, using different *T. thermophilus* strains^a^.

Strain	2’-Deoxyinosine synthesis			Inosine synthesis		
	Yield (%)	[cells ]^b^	Productivity^c^	Yield (%)	[cells ]^b^	Productivity^c^
NAR1	24	10,000	12 x 10^-5^	8.5	9,883	4.3 x 10^-5^
HB27	28	6,364	22 x 10^-5^	14	6,364	11 x 10^-5^
PRQ-16	24	6,316	19 x 10^-5^	11	6,111	9.0 x 10^-5^
PRQ-25	25	6,250	20 x 10^-5^	11	5,978	9.2 x 10^-5^
B	24	7,059	17 x 10^-5^	11	7,142	7.7 x 10^-5^
RQ1	20	10,417	9.6 x 10^-5^	8.7	10,610	4.1 x 10^-5^
N17	21	7,500	14 x 10^-5^	10.5	7,500	7.0 x 10^-5^
HN1.11	12	3,529	17 x 10^-5^	5.3	3,786	7.0 x 10^-5^
Fiji3A1	16.5	2,750	30 x 10^-5^	8.3	2,767	15 x 10^-5^
CC16	23	6,053	19 x 10^-5^	10	5,952	8.4 x 10^-5^
VG7	22	2,683	41 x 10^-5^	13	2,708	24 x 10^-5^

^a ^Reaction conditions: 65ºC, 5 mM deoxyuridine or uridine, 5 mM hypoxanthine, 30 mM sodium phosphate buffer (pH= 7). Total volume 4 mL; ^b ^million cells. mL^-1^;^ c ^µmol.h^-1^.million cells^-1^.

**Table 2:** Yield and productivity of the reaction dUrd or Urd + adenine using some selected strains ^a^.

Strain	2’-Deoxyadenosine synthesis			Adenosine synthesis		
	Yield (%)	[cells] ^b^	Productivity ^c^	Yield (%)	[cells] ^b^	Productivity ^c^
NAR1	30	10,714	14 x 10^-5^	15	10,563	7.1 x 10^-5^
HB27	44	11,000	20 x 10^-5^	21	10,938	9.6 x 10^-5^
PRQ-16	29	6,041	24 x 10^-5^	15	6,000	12._5_ x 10^-5^
PRQ-25	28	5,600	25 x 10^-5^	15	5,556	13._5_ x 10^-5^
B	38	9,500	20 x 10^-5^	21	9,545	11 x 10^-5^
RQ1	28	10,769	13 x 10^-5^	15	10,870	6.9 x 10^-5^
N17	27	7,105	19 x 10^-5^	12	6,771	9.6 x 10^-5^
HN1-11	15	5,769	13 x 10^-5^	6.9	5,847	5.9 x 10^-5^
Fiji3A1	14	5,000	14 x 10^-5^	6.3	5,250	6.0 x 10^-5^
CC16	30	6,818	22 x 10^-5^	14	6,667	10._5_ x 10^-5^
VG7	23	4,423	26 x 10^-5^	12	4,286	14 x 10^-5^

^a^ Reaction conditions: 65ºC, 5 mM deoxyuridine or uridine, 5 mM hypoxanthine, 30 mM sodium phosphate buffer (pH= 7). Total volume 4 m; ^b ^million cells. mL^-1^;^ c ^µmol.h^-1^.million cells^-1^.

In order to test the efficiency of the biocatalysts in the synthesis of 6-aminopurine nucleosides, the synthesis of 2’-deoxyadenosine (X=H) and adenosine (X=OH) (Scheme 2) was studied (Table 2). Productivity values, which are related to the number of cells carrying the reaction, were used to compare results, since each assay was performed from different cultures with different cells concentrations, making overall yield a poor parameter for comparison.

As in the case of 6-oxonucleosides (Table 1), the synthesis of 2’-deoxyadenosine (dAdo) leads to better yields than the synthesis of adenosine (Ado) (Table 2). The productivities in dAdo (Table 2) were generally similar or higher compared to those in the synthesis of 2’-deoxyinosine (Table 1) except for strains VG7 and FIJI3A1. In any case, adenine nucleoside degradation products (inosine, 2’-deoxyinosine or hypoxanthine) could not be detected, indicating absence of ADA activity at the tested temperature. From the results of Table 1 and Table 2, we could conclude that the intracellular enzymes of strains show better selectivity versus 6-aminopurine than versus 6-oxopurines. This broad specificity has been described in mesophile bacteria [5,7]. Due to the different biomass production of the strains, productivity values were calculated to select the best biocatalysts. According to this criterion HB27, PRQ-16, PRQ-25, B, Fiji3A1 and VG7 were selected as the most interesting strains. The productivity values are better than those described for wild type mesophile or psychrophile species under the same experimental conditions [5,6,8]. Other workers [9] describe better nucleoside yields but the cell concentration was not given, so we cannot compare the catalytic activity. These productivities, the absence of ADA activity, the thermotolerance and the resistance to extreme conditions make these biocatalysts interesting for further developments. In addition, we must indicate that the cell cultures of each strain – in optimum conditions – are repetitive as we can observe in Table 1 and Table 2.

**Table 3:** Yield and productivity of the reaction thymidine + adenine or hypoxanthine^a^.

Strain	Thymidine + hypoxantine			Thymidine + adenine		
	Yield (%)	[cells]^b^	Productivity^c^	Yield (%)	[cells]^b^	Productivity^c^
HB27	22	10,000	11 x 10^-5^	33	9,706	17 x 10^-5^
PRQ-16	14	5,385	13 x 10^-5^	21	5,526	19 x 10^-5^
PRQ-25	15	5,357	14 x 10^-5^	21	5,250	20 x 10^-5^
B	21	8,750	12 x 10^-5^	39.5	8,977	22 x 10^-5^
Fiji3A1	12	5,455	11 x 10^-5^	16	5,333	15 x 10^-5^
VG7	11.5	3,382	17 x 10^-5^	19	3,519	27 x 10^-5^

^a^Reaction conditions: 65ºC, 5mM thymidine, 5mM adenine or hypoxanthine, 30mM sodium phosphate buffer (pH=7). Total volume 4 mL; ^b ^million cells. mL^-1^;^ c ^µmol.h^-1^.million cells^-1^.

Similar reactions were performed using thymidine as the sugar-donor nucleoside and adenine or hypoxanthine as acceptor bases to explore the presence of one thymidine-nucleoside phosphorylase (selective *versus* thymidine and 2’-deoxyuridine (dUrd) compared to uridine (Urd) [11,12,13]). The combination of this enzyme plus a non-specific PNP can give activities similar to the expected for an active NdRT. The obtained productivity values with the selected strains (Table 3) were generally lower than those obtained with dUrd (Table 1 and Table 2) showing a moderated selectivity versus adenine. Therefore, if the strains: i) gave better yields versus adenine than with hypoxanthine; ii) gave better yields with 2’-deoxyribose nucleoside than with ribose nucleoside; iii) gave lower or similar yields using thymidine than using 2’-deoxyuracil, as sugar donors and iv) uridine is recognised as substrate, we could postulate that the strains present an active NdRT or one PyNP plus one PNP selective versus adenine compared to hypoxanthine. 

Other assays were performed for the optimization of reaction parameters such as pH, molar ratio of nucleoside/base and nature of the buffer. HB27 was selected due to the high productivities and the easy culture conditions. To explore the nature of the active enzyme, the same reaction was tested in two buffers. In Tris/HCl buffer 30mM (pH=7) the productivity values experienced an increase of 44% over those obtained in 30mM sodium phosphate buffer (pH=7). This result suggests, in accordance with the literature [1,2,3,4,6], that one NdRT, rather than two NPs, may be the most active enzyme in HB27.

**Scheme 3:**
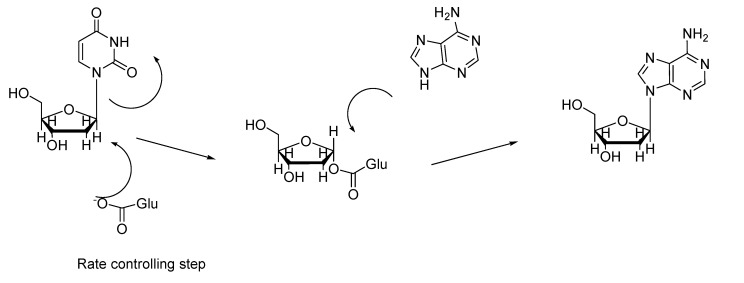
Reaction mechanism of the N-2-deoxyribosyltranferase catalysis.

**Figure 1:**
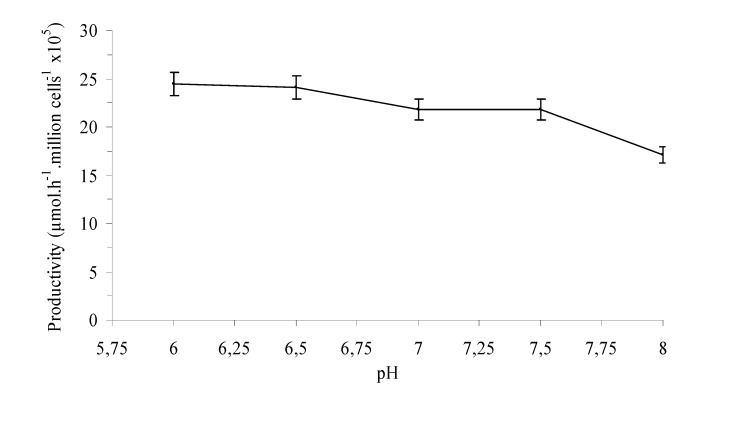
Influence of pH in the productivity of deoxyadenosine by *T. thermophilus* HB27. Standard reaction conditions: 65ºC, 5mM deoxyuridine, 5mM adenine, 30mM sodium phosphate buffer (pH=7). Total reaction volume 4 mL.

**Figure 2:**
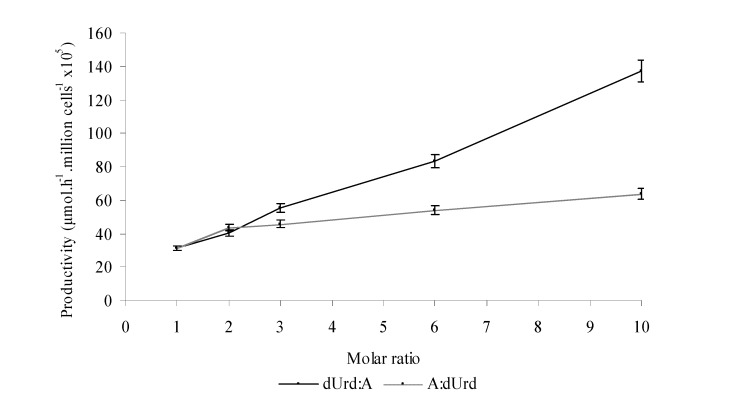
Influence of deoxyuridine/adenine ratio. Standard reaction conditions: 65ºC, 5mM deoxyuridine, 5mM adenine, 30mM sodium phosphate buffer (pH=7). Total reaction volume 4 mL.

In Scheme 3 we show the reaction mechanism for the 2’-deoxynucleoside synthesis catalyzed by NdRT [3,5]. We can see that phosphate ion is not necessary for the catalytic process**. **This is the fundamental difference with NPs, which produce 1-α-ribose-phosphate [5,11,14,15].

The results in Figure 1 indicate that better productivities are obtained at pH <7.5. Similar optimum pH values were recommended in the case of NdRTs from *Lactobacillus helveticus* (6 <pH <6.5 [16]) or *Lactococus, Streptoccocus, Aeroccoccus and Leuconostoc* genera (pH =6.5 [17]). Therefore, these results seem to support the NdRT hypothesis. 

In Figure 2 we show that an excess in dUrd dramatically increases the productivity values. Similar results have been described [5,9,16,18,19], suggesting that the rate controlling step is the first one (Scheme 3). The excess of adenine moderately increases the yield. Taking into the account the mechanism of NdRT [14,15] we could only explain this result by assuming that the greater the concentration of dUrd is, the higher the concentration of active intermediate (1-α-glutamyl-ribose , Scheme 3) is, and therefore the yield increases. Contrarily, if the dUrd concentration is the same, the concentration of the reaction intermediate is constant and so an increase in the adenine concentration does not dramatically increase the yield as observed (Figure 2). We must indicate that an increase in the adenosine concentration does not lead to hypoxanthine formation.

Several results hint towards the possibility of a NdRT involved in the reactions tested, such as the increased productivity when the reaction is carried in tris-HCl buffer without the presence of phosphate, which is a substrate in the NP reaction, or the apparent acidophility of the process. However, whole, living cells are a complex system with many variables which should be taken into the account. Also, it has been previously reported the existence of NPs in some of the tested strains, such as HB8 [20]. Now, cloning and isolation experiments are in progress in HB27 and PRQ-25 and VG7.

## Experimental

### Materials and Microorganisms

Nucleosides and purine bases were from Sigma-Aldrich (USA). HPLC solvents were from Scharlab (Spain) and the buffer reagents from Aldrich (Germany). Different strains of *Thermus thermophilus* were used in this work: NAR1, HB27, PRQ-16. PRQ-25, B, RQ1, Fiji3A1, HN1.11, CC16, VG7 and NR-17. Cells were grown in TB culture medium [0.8 % peptone (w/v), 0.4 % yeast extract (w/v) and 0.3 % NaCl (w/v) in Milli-Q grade water, adjusted to pH 7.5 with NaOH] at 65 ºC [7] for 16 h under shaking at 150 rpm in Erlenmeyer flasks. After growing, the culture broth was centrifuged for 15 min at 10,000 g. The cells were harvested and washed in 10 mL of 30 mM sodium phosphate buffer (pH =7) and then re-centrifuged. The pellet was directly used in the purine nucleoside synthesis test reactions.

### Standard synthesis of purine nucleosides

Cells were obtained from 20 mL of culture broth and resuspended 4 mL of reaction mixture (30 mM sodium phosphate buffer, pH=7, sugar-donor nucleoside 5 mM and sugar-acceptor base 5 mM). The reactions were performed at 65ºC under shaking for 1 hour. Samples were obtained and filtered to be immediately analysed by HPLC.

### Synthesis in Tris/HCl buffer

Cells were obtained from 20 mL of culture broth and resuspended with 4 mL of reaction mixture (30mM Tris/HCl buffer (pH=7)). Then 2’-deoxyuridine (5 mM) and adenine (5 mM) were added. The reaction was performed at 65ºC under shaking for 1 hour. Samples were filtered to be immediately analysed by HPLC. 

### Sample analysis

The samples were analyzed by HPLC using water/methanol (90:10 v/v) as the mobile phase and a flow rate of 1.2 mL·min^-1^. The Agilent 1100 Series HPLC was equipped with an UV detector (set at 254 nm) and a C_18_ apolar column (0.46 x 15 cm, 5 µm), Teknokroma, Barcelona, (Spain).
